# In Reply to Özkıdık et al.

**DOI:** 10.4274/balkanmedj.2018.1180

**Published:** 2018-07-24

**Authors:** Hasan Bozkurt, Serkan Şahin

**Affiliations:** 1 Department of Child and Adolescent Psychiatry, Gaziosmanpaşa University Hospital, Tokat, Turkey

To the Editor,

We read the letter that criticizes our previous report published in Balkan Medical Journal. Commentaries to the letter are as noted below respectively (1).

Firstly, information regarding the medical and psychiatric history of this child case was taken from mother and we unfortunately did not have the chance to observe the penil erection attacks in a clinical setting. So we reported this pediatric case based on the mother’s subjective observations. Those painless erections were only associated with olanzapine but not with any other factors or periods like day or night and resolved spontaneously after ceasing the treatment. According to mother, her son has never experienced such like these penil erections leading her to cease the drug. However we agree that information about the number of attacks should have been indicated in the case report. 

Secondly, we already stated that methylphenidate does not seem to cause priapism since the case benefited from methylphenidate without any side effect except loss of appetite. Naranjo algorithm for olanzapine rather than methylphenidate also classed the adverse reaction as “most probably” (1). Additionally, the case kept using methylphenidate while priapism resolved after ceasing olanzapine.

The commonest causes of priapism in children are reported to be sickle cell disease (65%), leukaemia (10%), trauma (10%), idiopathic (10%), and pharmacologically induced (5%) (2). We excluded these conditions with medical history and blood work in the present case. However radiological evalution like colour duplex ultrasonography is usually helpful for distinguishing ischaemic and non-ischaemic priapism during attacks and also helpful in guiding treatment. As stated above, we could not directly observe the prapism attacks, so radiological examination was not thought to make a contribution on diagnosis and treatment of our case report (3).
